# Description of a National Cohort of Patients With Left Atrial Appendage Occlusion Devices

**DOI:** 10.7759/cureus.91618

**Published:** 2025-09-04

**Authors:** Zhe Chen, Bryan G Kane

**Affiliations:** 1 Department of Emergency and Hospital Medicine, University of South Florida Morsani College of Medicine, Lehigh Valley Health Network, Bethlehem, USA

**Keywords:** atrial fibrillation, laao, risk stratification, stroke, watchman

## Abstract

Background

Stroke is a known complication of atrial fibrillation. To avoid anticoagulation therapy, left atrial appendage occlusion (LAAO) devices are often placed in patients.

Objective

This study, via a national cohort of patients, sought to describe the risks associated with LAAO.

Methods

Data used in this study came from Epic Cosmos, a dataset created in collaboration with a community of Epic health systems representing more than 296 million patient records from all 50 states, Washington D.C., Lebanon, and Saudi Arabia. Using this dataset, we assembled a cohort of patients who had an LAAO placed. Demographic information was gathered to risk-stratify via the Congestive heart failure, Hypertension, Age ≥75 years, Diabetes mellitus, Stroke, Vascular disease, Age 65-74 years, Sex category (CHA_2_DS_2_-VASc) score. Diagnosis of stroke was made using visits with that diagnosis after LAAO placement. Descriptive statistics and hazard ratios were calculated. This cohort was compared to previously published ones.

Results

The study cohort assembled consisted of 51,682 patients who underwent implantation of an LAAO from 2021 to 2023. The average age was significantly higher than previously published (p < 0.001), as was the risk of stroke. CHA_2_DS_2_-VASc of 7 or greater was associated with elevated risk of stroke after LAAO. For CHA_2_DS_2_-VASc of 7-9, the hazard ratio ranged from 4.88-6.32, with significant 95% confidence intervals.

Conclusions

This cohort suggests that the risk of stroke after LAAO implantation is higher than previously reported, though findings are limited by their retrospective nature. Patients with elevated CHA_2_DS_2_-VASc scores may require additional anticoagulation therapy to reduce the statistically significant increased risk of stroke. CHA_2_DS_2_-VASc scores, in this cohort, appear to stratify patients' stroke risk after LAAO placement.

## Introduction

Atrial fibrillation (Afib) is the most common sustained cardiac dysrhythmia and cause of morbidity and mortality in the world [1]. Caused by irregular, ectopic electrical initiation, Afib has both direct and indirect causes of harm [2]. Examples of direct harm include heart failure via Afib-induced rapid ventricular response or the absence of an atrial kick [3]. Afib is an independent risk for coronary artery disease and may potentiate ventricular dysthymias [2]. Thromboembolic stroke, which typically requires initiation of anticoagulation (AC) therapy, is strongly associated with Afib due to blood stasis in the left atrial appendage and left atrium [4]. This serves as an example of both direct and indirect harm, as AC carries with it its own risk [5]. Various risk stratification systems have been developed to guide clinician prescription of AC for Afib, though the Congestive heart failure, Hypertension, Age ≥75 years, Diabetes mellitus, Stroke, Vascular disease, Age 65-74 years, Sex category (CHA_2_DS_2_-VASc) system is broadly endorsed [6-10].

More recently, alternatives to AC therapy have been developed. Within the heart, the left atrial appendage is a small outpouching of the muscular wall of the left atria. With the secretion of natriuretic peptides, the left atrial appendage is part of the complex system which monitors and regulates intravascular volume [11]. Prior research has determined that nearly all, 91% to 100%, thrombus formation occurs in the left atrial appendage [12]. As an alternative to AC, left atrial appendage occlusion devices (LAAO) have been developed to mitigate stroke risk [13]. Investigations to compare LAAO to AC have been published, though the question’s magnitude may not be accurately represented by cohort sizes in those studies [14-16].

Patients who receive care at an institution using the electronic medical record system Epic (Epic Systems, Verona, WI, USA) may volunteer to have information entered into Cosmos [17]. In total, Cosmos has over 276 million patients with 13.8 billion encounters [18]. The process of using Cosmos for investigation has been previously described [19]. This study utilized Cosmos to develop a large, national cohort of patients with LAAO placement and describe their baseline risk for, and development of, subsequent stroke.

## Materials and methods

Data were extracted from Epic Cosmos, creating a “Cosmos cohort.” This dataset was created by Epic Systems and represents more than 296 million patient records from over 41,000 hospitals and clinics throughout the United States, Lebanon, and Saudi Arabia [20]. For patient protection, Epic de-identifies and date shifts all information at the patient level before it is included in the Cosmos dataset. The Cosmos dataset has been reviewed by the University of Wisconsin, which describes this type of large dataset work as being exempt by the Federal Common Rule [21]. This study was reviewed and approved by our independent, internal IRB.

To assemble our cohort of patients, presence of an LAAO device was defined as a patient who both had a documented billed procedure Current Procedural Terminology code of 33340 (percutaneous transcatheter closure of the left atrial appendage with implant) and had a documented procedure end date. The procedure end date allowed for determination of whether a stroke occurred prior to or following LAAO implantation. Only those patients with an LAAO Current Procedural Terminology code 33340 procedure performed between 1/1/2021 and 12/31/2023 were included. This was defined as patients having the coded or charged procedure code and a documented procedure end date and time for the procedure. This date range was chosen for efficient data querying to obtain a dataset representative of a recent cohort with a time frame to allow post-procedure follow-up. In order to measure subsequent stroke risk, only patients with continued follow-up documented in Cosmos, defined as two office visit encounters post-procedure, were included. A patient was considered to have had a stroke post-procedure if there was a documented hospital admission with a Stroke International Classification of Diseases 10th revision (ICD-10) code as the admitting, discharge, encounter, or billing diagnosis with a more recent date than the LAAO placement. Transient ischemic attack was included as a part of the definition of a stroke event. No missing data handling was needed for age, race, or gender. For diagnoses, any patient who did not have data for a particular diagnosis was assumed to not have the diagnosis. Medical histories of hypertension, diabetes mellitus, vascular disease, stroke, transient ischemic attack, thromboembolism, and congestive heart failure were extracted if their respective ICD-10 codes were documented on the patient’s problem list before the LAAO procedure date. A complete list of extracted ICD-10 codes is found in Appendix 1.

Demographics including age, race, and sex, along with procedure dates, post-procedure stroke admission dates, and past medical history were extracted using Structured Query Language (SQL) to create a dataset for analysis. The SQL query was written and created in the data science virtual machine hosted by Epic, a purpose-built tool designed to conduct research in Epic’s Cosmos dataset. The first SQL query was created to pull patient demographic information and procedure date-time where documented procedures of LAAO happened between 1/1/2021 and 12/31/2023. Subsequently, queries of diagnoses relevant to CHA_2_DS_2_-VASc scoring were run, where the diagnosis must have occurred prior to the procedure date, and each was added to the LAAO initial dataset using the Left Join SQL function. Following this, the target diagnosis table was created, which involved a patient encounter occurring after the procedure date, with an admitting, discharge, billing, and final diagnosis with a stroke ICD code. This table was then joined onto the initial LAAO dataset.

The CHA_2_DS_2_-VASc score was calculated using extracted variables [6-10]. To calculate the CHA_2_DS_2_-VASc score, each patient’s age was calculated using the procedure time and patient’s birthday. The age was then binned. For diagnoses relevant to the score, such as congestive heart failure, diabetes, history of stroke, stroke, transient ischemic attack, thromboembolism history, and vascular disease history, the relevant diagnosis was only included and counted in the calculation if it was listed on the patient’s problem list prior to the procedure. Risk of stroke within one year post-LAAO implantation was stratified by CHA_2_DS_2_-VASc score. Statistical analyses were conducted using R (R Foundation for Statistical Computing, Vienna, Austria) and RStudio (Version 4.4.1; Posit PBC, Boston, MA, USA). Cox hazard survival analysis was performed using the survival package in R. We then compared the Cosmos cohort to previously published studies of LAAO, including the PRAGUE-17, PREVAIL, and PROTECT AF cohorts [14-16].

## Results

In total, 51,682 patients who underwent implantation of an LAAO were included for analysis. Of these, 2,054 patients (4%) had a diagnosis of new stroke post-procedure. The demographic and clinical characteristics of patients with and without stroke outcomes are presented in Table 1. In 66% (N=1,347) of patients who had a stroke after their LAAO, there was a preexisting history of stroke, transient ischemic attack, or thromboembolism at the time of their procedure. This history was present in 32% (N=15,719) of patients who did not have a post-procedure stroke (Table 1).

**Table 1:** Cohort demographics and pre-procedure medical history ^†^Values are Mean ± Standard Deviation or n (%). Total n = 51,682 patients. LAAO: left atrial appendage occlusion

Characteristic	No Stroke Documented After LAAO (N = 49,628)	New Post-Procedure Stroke Documented (N = 2,054)
Age		
Mean Age (Years)	76.73 ± 7.73^†^	77.48 ± 7.67
Age Over 74	31,740 (64%)^†^	1,397 (68%)
Age 65 to 74	15,004 (30%)	551 (27%)
Sex		
Female	20,644 (42%)	864 (42%)
Male	28,982 (58%)	1,190 (58%)
Race		
American Indian or Alaska Native	266 (0.5%)	11 (0.5%)
Asian	511 (1.0%)	23 (1.1%)
Black or African American	2,162 (4.4%)	161 (7.8%)
Native Hawaiian or Other Pacific Islander	69 (0.1%)	2 (<0.1%)
Other Race	343 (0.7%)	14 (0.7%)
White	46,140 (93%)	1,839 (90%)
Pre-Procedure Medical History		
Hypertension	47,461 (96%)	1,996 (97%)
Vascular Disease	35,830 (72%)	1,593 (78%)
Diabetes Mellitus	21,359 (43%)	1,044 (51%)
Prior Stroke/Transient Ischemic Attack/Thromboembolism	15,719 (32%)	1,347 (66%)
Congestive Heart Failure	27,805 (56%)	1,289 (63%)

**Figure 1:**
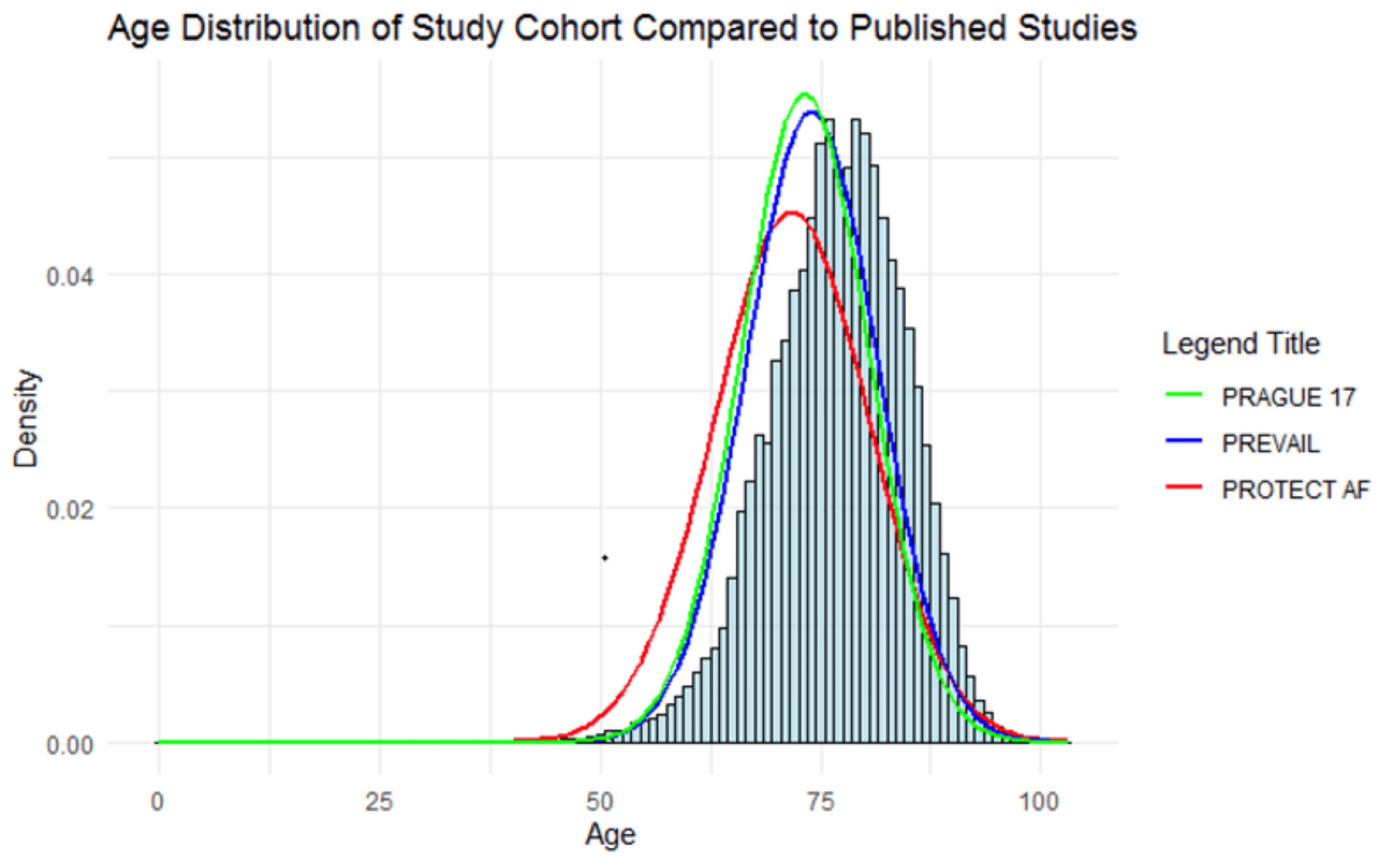
A comparison of patient ages found in the Cosmos LAAO Cohort (light blue bars) to the PRAGUE-17 (green line), PREVAIL (blue line), and PROTECT AF (red line) studies. Each bin represents a single year of age. The mean age of this cohort was significantly older than previously published trials (one-sample t-test; 76.73 years; 95% CI, 76.70-76.83, t = 2,257; p < 0.001). PRAGUE-17, PREVAIL, PROTECT AF: [14-16] LAAO: left atrial appendage occlusion

The age distribution of the study cohort is presented in Figure 1, which also compares the age distribution of our cohort with those from the PRAGUE-17, PREVAIL, and PROTECT AF cohorts. The comparison was created using the published means and standard deviations for age [14-16]. Figure 1 demonstrates that the cohort of LAAO patients assembled from Cosmos is, with statistical significance, older compared to the published mean ages in those three clinical trials (one-sample t-test; 76.73 years; 95% CI, 76.70-76.83; t = 2,257; p < 0.001).

The incidence of strokes in the Cosmos cohort was higher at 3.1% (N = 51,682) events/year than previous results reported in the PREVAIL (1.5% [N = 269] events/year), PRAGUE-17 (1.9% [N = 201]), and PROTECT AF (2.6% [N = 244] events/year) studies [14-16]. Of note, in the PREVAIL trial, the stroke rate in the device arm was presented as an overall percentage. Due to heterogeneous definitions of strokes in these cohorts, no tests of significance were performed.

**Figure 2:**
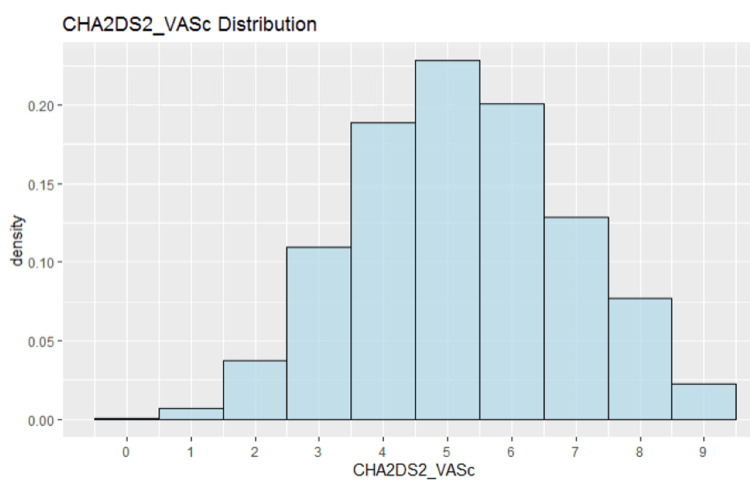
The distribution of CHA2DS2-VASc scores across the entire cosmos cohort. Bins represent a single score value, with scores 4-6 being the most common. CHA_2_DS_2_-VASc: Congestive heart failure, Hypertension, Age ≥75 years, Diabetes mellitus, Stroke, Vascular disease, Age 65-74 years, Sex category

The distribution of CHA_2_DS_2_-VASc scores among patients in the Cosmos cohort of patients with LAAO device implantation is shown in Figure 2. The most common scores in this cohort were 4-6. Table 2 demonstrates the rate of stroke in patients stratified by their CHA_2_DS_2_-VASc score. Percentages reported are of the entire cohort. Hazard ratios for a post-LAAO stroke were calculated to assess the risk stratification provided by the CHA_2_DS_2_-VASc scores. Those ratios are shown in Table 3. At a score of 7 or greater, there is a significantly higher risk of stroke post-LAAO procedure.

**Table 2:** Occurrence of stroke stratified by CHA2DS2-VASc distribution Values are n (%). Total n = 51,682 patients. LAAO: left atrial appendage occlusion, CHA_2_DS_2_-VASc: Congestive heart failure, Hypertension, Age ≥75 years, Diabetes mellitus, Stroke, Vascular disease, Age 65-74 years, Sex category

CHA_2_DS_2_-VASc Score	No Stroke Documented After LAAO (N = 49,628)	New Post Procedure Stroke Documented (N = 2,054)
0	20 (<0.1%)	0 (0%)
1	179 (0.4%)	3 (0.1%)
2	1,398 (2.8%)	16 (0.8%)
3	5,082 (10%)	80 (3.9%)
4	9,460 (19%)	199 (9.7%)
5	11,655 (23%)	334 (16%)
6	10,338 (21%)	452 (22%)
7	6,468 (13%)	501 (24%)
8	3,871 (7.8%)	356 (17%)
9	1,157 (2.3%)	113 (5.5%)

**Table 3:** Risk of stroke by CHA2DS2-VASc score ^†^CI = Confidence Interval Results were considered significant when p<0.05 CHA_2_DS_2_-VASc: Congestive heart failure, Hypertension, Age ≥75 years, Diabetes mellitus, Stroke, Vascular disease, Age 65-74 years, Sex category

CHA_2_DS_2_-VASc Score	Hazard Ratio	95% CI^†^	p-value	Z-statistic
1				
2	0.76	0.22 - 2.59	0.7	-0.446
3	1.05	0.33 - 3.32	>0.9	0.079
4	1.42	0.45 - 0.44	0.5	0.602
5	1.95	0.63 - 6.08	0.2	1.152
6	2.87	0.92 - 8.92	0.069	1.817
7	4.88	1.57 - 15.20	0.006	2.736
8	5.96	1.91 - 18.60	0.002	3.078
9	6.32	2.01 - 9.90	0.002	3.152

The one-year risk of stroke post-LAAO for the Cosmos cohort was stratified by CHA_2_DS_2_-VASc scores and compared to previously published risk of ischemic stroke for patients without warfarin from the Swedish Afib cohort (Table 4) by Friberg et al. [22]. There is a significant increase in stroke risk from a score of 7 up to 9 when compared to baseline score of 1 using Cox proportional hazards survival analysis (range from p < 0.002 to < 0.006).

**Table 4:** Risk of stroke at one year post-LAAO procedure for cosmos and Swedish Afib cohorts Values are represented as percents of all patients with the corresponding risk scores who experienced a stroke <1 year following LAAO implantation. LAAO: left atrial appendage occlusion, Afib: atrial fibrillation Swedish Afib cohort: Friberg et al. [22]

CHA_2_DS_2_-VASc Score	Cosmos Cohort	Swedish Cohort
1	- -	0.60%
2	0.81%	2.20%
3	1.13%	3.20%
4	1.52%	4.80%
5	2.08%	7.20%
6	3.05%	9.60%
7	5.13%	11.20%
8	6.23%	10.80%
9	6.60%	12.20%

## Discussion

This Cosmos cohort of 51,682 patients is the largest cohort of LAAO patients assembled to date. The dataset’s size and, subsequently, the size of the LAAO patient cohort allows for analysis of patient outcomes with an unprecedented level of power. During the COVID-19 pandemic, Cosmos was used to identify associations between the virus and myocarditis [17]. More recently, Cosmos has been used to better describe the presentation of over a million patients with congestive heart failure to emergency departments around the United States [23]. The same author has been able to rapidly publish evaluations of emergency department presentations for diverticulitis and pulmonary embolism [24,25]. Our work on this cohort serves as a sort of external validation to this early work in Cosmos.

Our cohort differs fundamentally from prior published work in that it was significantly older, and the risk of stroke was higher, than the PRAGUE-17, PREVAIL, and PROTECT AF cohorts [14-16]. Regarding predictive use of CHA_2_DS_2_-VASc, our findings are consistent with those prior studies, in that they do appear to support the score’s utility. The Cosmos cohort demonstrates a clear increased risk of stroke as the CHA_2_DS_2_-VASc score increases. Conversely, our cohort appears to support the concept that patients with low CHA_2_DS_2_-VASc scores appear to be low risk for stroke after LAAO implantation. Of concern though, is our observation that at higher CHA_2_DS_2_-VASc scores, a significant risk of stroke remains, despite the presence of a LAAO device.

There are clear limitations to the Cosmos dataset, many of those related to those traditionally associated with retrospective chart reviews [26]. It should also be noted that social determinants of health may significantly alter patients’ cardiovascular risk [27]. This may impact the generalizability of our results, as social factors such as access to care or socioeconomic status are not adequately tracked within the Cosmos dataset. Additionally, significance testing when comparing this cohort to previously published studies was not possible due to heterogeneous definitions of stroke.

Further research is needed to determine if anti-platelet or AC therapy is warranted in conjunction with LAAO in the face of elevated CHA_2_DS_2_-VASc scores. Finally, the Cosmos cohort could be expanded to include patients with AFib on AC, allowing a much larger comparison than recently published cohorts [28]. Alternatively, it may be possible to follow emerging types of LAAO with larger numbers [29].

## Conclusions

While limited by the retrospective nature of the study, this cohort suggests that the risk of stroke after LAAO implantation is higher than previously reported. Patients with elevated CHA_2_DS_2_-VASc scores may require additional AC to reduce the statistically significant increased risk of stroke observed. CHA_2_DS_2_-VASc scores, in this cohort, appear to risk-stratify patients' stroke risk after LAAO placement. This study constitutes the largest cohort of patients with LAAO implants to date. This unprecedented level of power enabled more precise analysis of the relationship between CHA_2_DS_2_-VASc score and risk of stroke than previous studies have provided.

## Appendices

**Table 5:** ICD-10 codes used as extraction and inclusion criteria to assemble the study cohort from Cosmos. ICD-10: International Classification of Diseases 10th revision, TIA: transient ischemic attack, CHF: congestive heart failure

Diagnosis	All ICD-10 codes That Start With:
Ischemic Stroke	I63 – Cerebral Infarction
Hypertension	I10 – Essential (primary) hypertension; I11 – Hypertensive heart disease; I12 – Hypertensive chronic kidney disease; I13 – Hypertensive heart and chronic kidney disease; I15 – Secondary hypertension; I16 – Hypertensive crisis; I1A – Other hypertension
Diabetes Mellitus	E08 – Diabetes mellitus due to underlying condition; E09 – Drug or chemical induced diabetes mellitus; E10 – Type 1 diabetes mellitus; E11 – Type 2 diabetes mellitus; E13 – Other specified diabetes mellitus
Vascular Disease	I73 – Peripheral vascular diseases; I70.2 – Atherosclerosis of native arteries of the extremities; I70.3 – Atherosclerosis of unspecified type of bypass graft(s) of the extremities; I70.4 – Atherosclerosis of autologous vein bypass graft(s) of the extremities; I70.5 – Atherosclerosis of nonautologous biological bypass graft(s) of the extremities; I70.6 – Atherosclerosis of nonbiological bypass graft(s) of the extremities; I70.7 – Atherosclerosis of other type of bypass graft(s) of the extremities; I21 – Acute myocardial infarction; I22 – Subsequent ST elevation (STEMI) and non-ST elevation (NSTEMI) myocardial infarction; I23 – Certain current complications following ST elevation (STEMI) and non-ST elevation (NSTEMI) myocardial infarction (within the 28 day period); I24 – Other acute ischemic heart diseases; I25 – Chronic ischemic heart disease
Prior Stroke, TIA, Thromboembolism	G45 – Transient cerebral ischemic attacks and related syndromes; G46 –Vascular syndromes of brain in cerebrovascular diseases; I63 – Cerebral infarction; I74 – Arterial embolism and thrombosis; I75 – Atheroembolism; I82.2 – Embolism and thrombosis of vena cava and other thoracic veins; I82.4 – Acute embolism and thrombosis of deep veins of lower extremity; I82.5 – Chronic embolism and thrombosis of deep veins of lower extremity; I82.6 – Acute embolism and thrombosis of veins of upper extremity; I82.7 – Chronic embolism and thrombosis of veins of upper extremity; I82.8 – Embolism and thrombosis of other specified veins; I82.A – Embolism and thrombosis of axillary vein; I82.8 – Embolism and thrombosis of subclavian vein; I82.C – Embolism and thrombosis of internal jugular vein; I26 – Pulmonary embolism
CHF	I42 – Cardiomyopathy; I43 – Cardiomyopathy in diseases classified elsewhere; I50 – Heart failure
